# Emerging therapeutic potential of umbilical cord-derived extracellular vesicles in lung-injurious diseases: a review of recent advances

**DOI:** 10.1186/s13287-026-05039-9

**Published:** 2026-05-12

**Authors:** Shuzhe Xiao, Lingling Wang, Xu Li, Qi Zheng, Lewen Zhou, Xiaoyan Song, Jie Yang

**Affiliations:** 1 Department of Neonatology, Nanfang Hospital, Southern Medical University, Guangzhou, 510515 China; 2 The First Clinical Medical College of Southern Medical University, Guangzhou, 510515 China; 3 Department of Neonatology, Guangdong Women and Children Hospital, Guangzhou, 511442 China; 4 Guangzhou Women and Children′s Medical Center, Guangzhou Medical University, Guangzhou, 510623 China

**Keywords:** Extracellular vesicles, Umbilical cord, Lung injury, Therapeutic potential, Engineering

## Abstract

Recent research has shown that extracellular vesicles (EVs) have significant potential in treating lung injuries. These vesicles facilitate communication between cells and regulate important biological processes, including cell growth, blood vessel formation, and inflammatory responses. Due to their natural ability to cross biological barriers and low risk of immune rejection, EVs are potential vehicles for targeted drug delivery. Among various biological sources, umbilical cord-derived EVs are highly advantageous because the collection process is non-invasive and provides a high yield of vesicles. Preclinical studies have demonstrated their therapeutic potential in conditions such as bronchopulmonary dysplasia, chronic obstructive pulmonary disease, acute respiratory distress syndrome, and asthma. This review summarizes the current evidence supporting the use of umbilical cord-derived EVs for lung diseases. It also discusses key translational challenges, such as manufacturing scalability and product consistency, alongside advanced engineering strategies for future clinical use.

## Introduction

Lung injury diseases, encompassing conditions such as acute respiratory distress syndrome (ARDS), chronic obstructive pulmonary disease (COPD), pulmonary fibrosis (PF), and bronchopulmonary dysplasia (BPD), represent a significant global health burden with limited therapeutic options and high morbidity and mortality.

Research in regenerative medicine is moving from cell transplantation toward the use of secreted factors, and extracellular vesicles (EVs) are currently the most prominent among these. Extracellular vesicles have garnered significant attention due to their remarkable biological diversity. These EVs deliver complex mixtures of proteins, lipids, and nucleic acid substances like microRNAs (miRNAs) and messenger RNAs (mRNAs), in which they transport things from one cell to another, playing an important role in the process, helping tissue repair to proceed smoothly. These include immune regulation, angiogenesis, and cellular reprogramming [1]. Compared to whole-cell therapies, EVs offer multiple advantages, including decreased immunogenicity, enhanced biocompatibility, and superior penetration of biological barriers. However, the biological source of EVs is a significant determinant of their efficacy and is sometimes overlooked. The therapeutic payload of EVs and their derived biological functions are fundamentally contingent upon the physiological state and type of the parent cell. It is within this context that umbilical cord (UC) emerges as a uniquely potent source. Rich in mesenchymal stromal cells (MSCs), hematopoietic stem cells (HSCs), and immunoregulatory cell types, UC is both ethically compliant and widely accessible as a biological material [2]. EVs derived from umbilical cord (UC-EVs) naturally inherit many of the regenerative and immunomodulatory traits of their parent cells [3]. These properties make EVs suitable for targeted therapy in respiratory diseases, as these conditions often require the modulation of immune responses and the promotion of tissue repair.

**Fig. 1:**
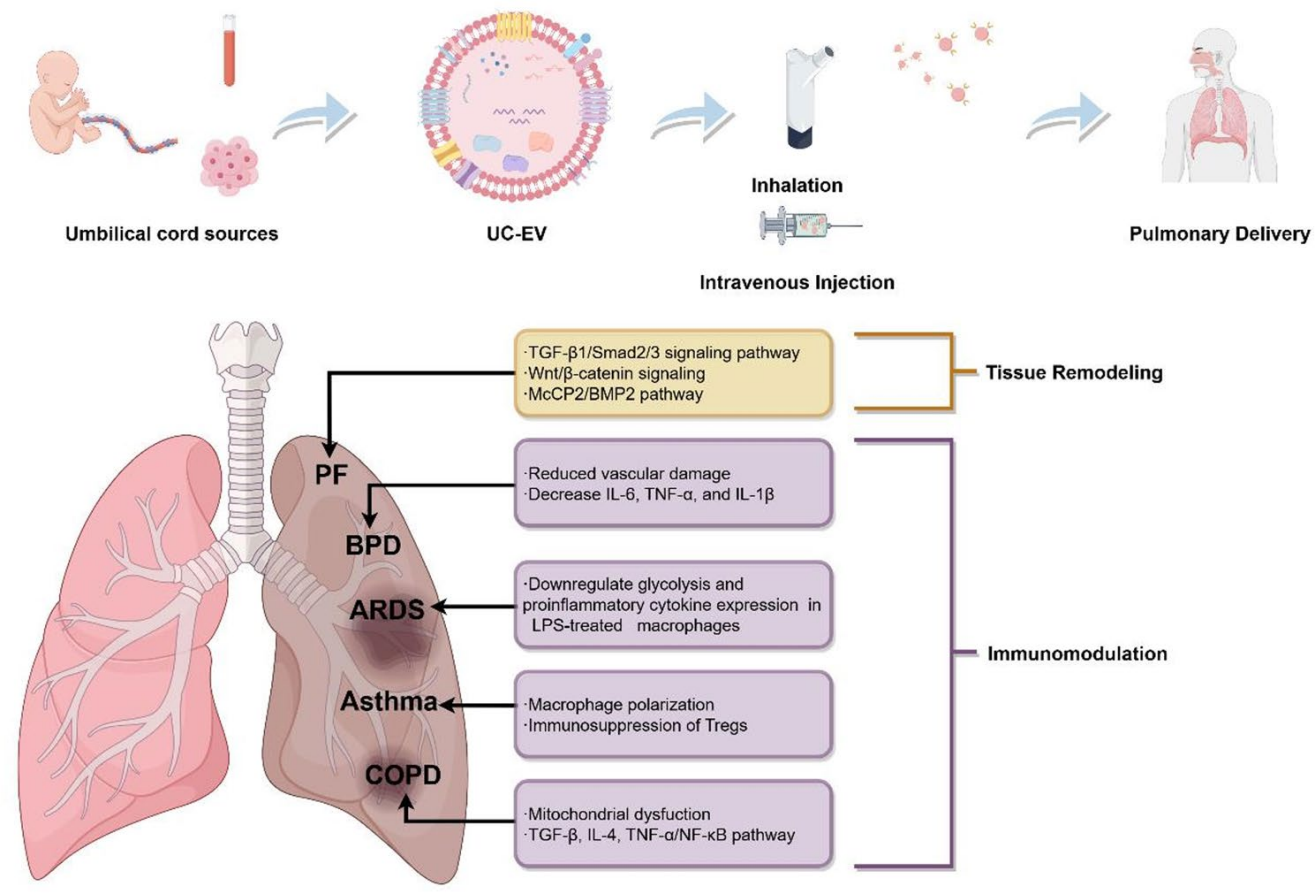
General therapeutic framework and molecular mechanisms of UC-EVs in lung-injurious diseases. The top panel illustrates the integrated process from biological sources to pulmonary delivery. UC-EVs are obtained from tissue or blood sources and administered via nebulized inhalation or intravenous injection. The bottom panel provides a comparative view of the therapeutic impact, contrasting a healthy repaired lung with various injured states. Specific molecular mechanisms for conditions such as PF, BPD, ARDS, asthma, and COPD are detailed. These mechanistic pathways are organized into two functional hierarchies: tissue remodeling, which focuses on structural repair, and immunomodulation, which involves the regulation of inflammatory cytokines and immune cell activity. This figure is original and was created by the authors using Figdraw (www.figdraw.com)

This review critically evaluates the therapeutic mechanisms of UC-EVs in lung injury, specifically analyzing how their cellular origin defines distinct functions and confronting the major challenges in clinical translation. The overall therapeutic framework of umbilical cord-derived EVs, including their biological sources, clinical delivery routes, and primary molecular mechanisms, is summarized in Fig. 1.

## Nomenclature and biological sources of umbilical cord-derived EVs

To ensure scientific accuracy and consistency across different studies, this review follows the Minimal Information for Studies of Extracellular Vesicles 2023 (MISEV2023) guidelines [4]. Although the term “exosome” is frequently used in previous literature, it refers specifically to vesicles formed through the endosomal pathway. Since this biogenesis pathway is difficult to prove in most therapeutic studies, we use the broader term “extracellular vesicles” (EVs) throughout this paper. Specifically, we focus on small EVs (sEVs), which are vesicles typically smaller than 200 nm.

**Fig. 2:**
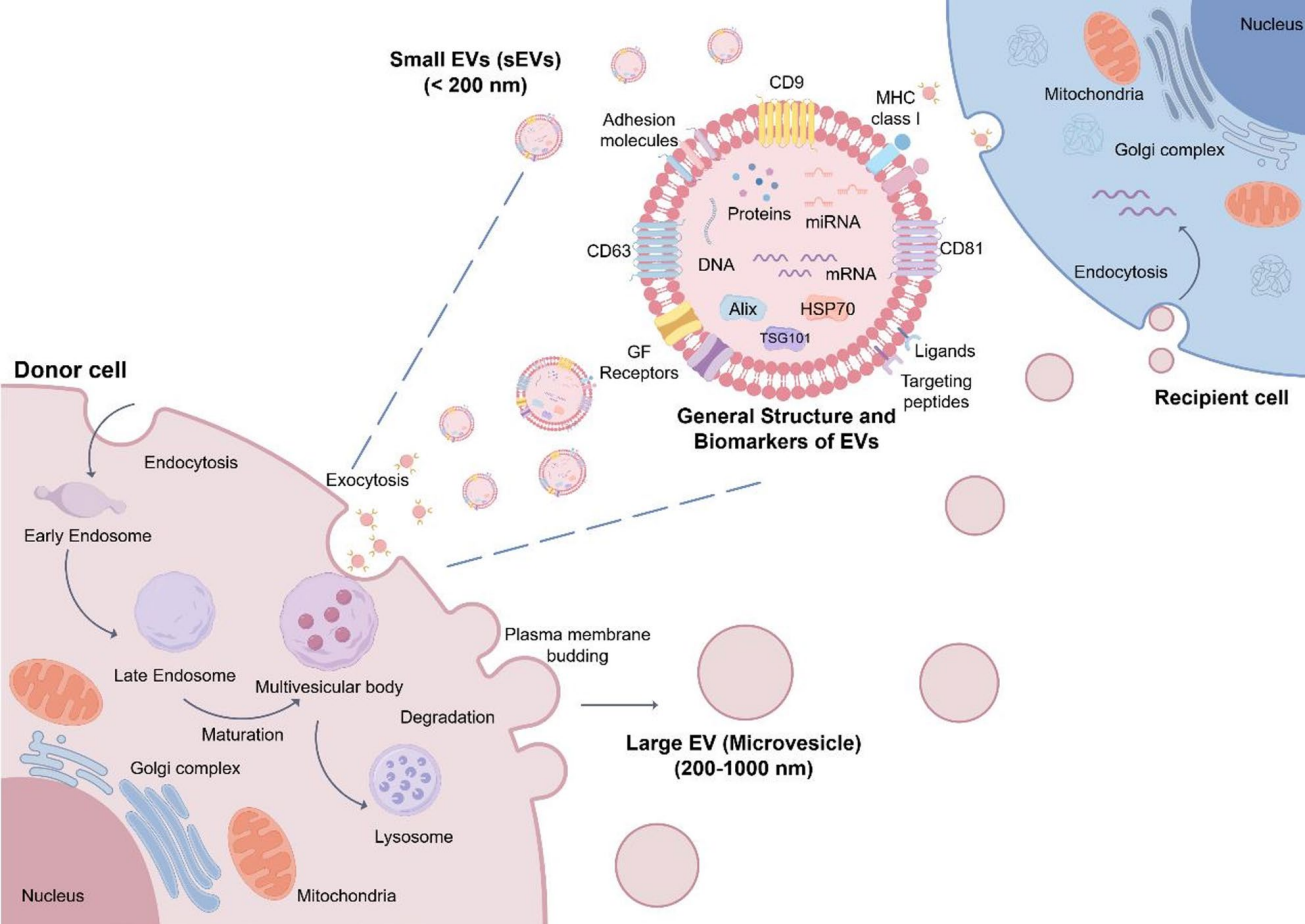
Biogenesis, molecular structure, and cellular interaction of UC-EVs. This figure depicts the complete life cycle of UC-EVs. In the donor cell (left), EVs are produced via two distinct routes: the endosomal pathway, involving the maturation of early and late endosomes into MVBs containing intraluminal vesicles and subsequent exocytosis of small EVs (sEVs, < 200 nm); and the direct plasma membrane budding pathway which generates large EVs (lEVs, 200–1000 nm). The magnified view (center) details the UC-EV structure, highlighting essential surface biomarkers (CD9, CD63, CD81, MHC class I) and internal cargo (miRNAs, mRNAs, proteins) including characterization markers like Alix and TSG101. The recipient cell interaction (right) illustrates the uptake of EVs and the release of therapeutic cargo to promote lung repair. The illustration was independently developed by the authors using the Figdraw platform (www.figdraw.com)

It is also necessary to clarify the different biological sources of these vesicles, as they are often mixed up in perinatal research. In this review, we categorize the evidence into two main groups. The first group is UC-MSC-derived EVs (UC-MSC-EVs), which are collected from the culture media of MSCs found in umbilical cord tissue. These are common in research because they are easy to produce in large amounts and have strong immune-regulating effects. The second group is umbilical cord blood-plasma EVs (UCBP-EVs), which are isolated directly from UCB plasma. By separating these sources and ensuring that each cited study uses standard characterization methods, such as nanoparticle tracking analysis, electron microscopy, and protein marker detection, this review provides a clear and reliable summary of the field. The biogenesis pathways of small and large EVs, their molecular structure, and their subsequent interaction with recipient cells are illustrated in Fig. 2.

### Biogenesis and universal characteristics of sEVs

Cells secrete heterogeneous populations of EVs, classified by size and biogenesis pathway. Small EVs (sEVs, 30–150 nm) originate from endosomal multivesicular bodies, while large EVs (lEVs), such as microvesicles bud directly from the plasma membrane. Apoptotic bodies (1–4 μm) are enriched in phosphatidylserine [4]. The formation of EVs commences deep within the cell, originating from the invagination of the early endosomal membrane. This invagination process forms multivesicular bodies (MVBs), essentially endosomal compartments filled with intraluminal vesicles (ILVs). This process is not random but is rigorously regulated by a molecular machinery collectively called the endosomal sorting complex required for transport (ESCRT) [5, 6]. Pathways independent of ESCRT (such as the ceramide-driven pathway) also contribute to ILV formation [7]. Once formed, multivesicular bodies ultimately fuse with the plasma membrane, releasing their contents into the extracellular environment. This final step is primarily regulated by Rab GTPases and SNARE complexes [8, 9]. As ubiquitous intercellular messengers, EVs carry a characteristic set of conserved membrane proteins (such as the transmembrane proteins CD9, CD63, and CD81) and encapsulate diverse cargo, including nucleic acids (miRNA, mRNA), lipids, and cytoplasmic proteins, which are selectively packaged to reflect the physiological state of the parent cell [10, 11].

### Mechanisms of target cell engagement

EVs primarily influence target cells through two mechanisms, although these often overlap. The first involves surface signaling, wherein direct interactions between ligands and receptors on the cell membrane activate downstream pathways without requiring internalization. The second mode involves cargo delivery, whereby EVs are taken up by recipient cells through processes such as endocytosis, phagocytosis, or even direct membrane fusion. Once inside the cell, the vesicles release their molecular contents, enabling these substances to alter cellular behavior or gene expression [12, 13]. The transmission pathways and ultimate effects of EVs are highly context-dependent. Factors such as the molecular composition of the EV outer layer and the physiological state of the recipient cell play decisive roles. This implies that the same EVs may trigger a signaling cascade in one cell type, yet be internalized and exert entirely different functions in another.

### Minimal characterization standards for research rigor

To ensure scientific rigor and reproducibility, the characterization of UC-EVs must follow the updated standards established by the MISEV2023 guidelines [4]. Proper identification requires the combination of several complementary analytical methods. First, physical properties such as particle size distribution and concentration are measured using nanoparticle tracking analysis, while the characteristic morphology is confirmed via transmission electron microscopy [14]. Beyond physical traits, biochemical analysis is necessary to verify the presence of specific protein markers. These include positive markers enriched in EVs, such as tetraspanins (CD9, CD63, and CD81) and cytosolic proteins like Alix and TSG101 [15]. Furthermore, demonstrating the absence of non-EV contaminants, such as calnexin or albumin, is essential to confirm the purity of the isolates [16]. Following these rigorous characterization standards ensures that experimental results are reliable and comparable across different studies.

Beyond these technical standards, the functional characteristics of UC-EVs are also fundamentally determined by their origin. Each vesicle carries a complex mixture of RNA, proteins, and lipids precisely molded by the parent cell’s properties, which determines its therapeutic potential [17, 18]. This source-dependent bioactivity makes UCB a particularly attractive source for regenerative medicine. This source contains many neonatal cells with immunomodulatory functions, providing a valuable basis for pulmonary repair and immune homeostasis.

## Biological sources and diversity of umbilical cord-derived EVs

UC contains various therapeutic components that are classified into tissue-resident and blood-derived elements. Umbilical cord tissue, specifically Wharton’s jelly, is the primary source of mesenchymal stromal cells (UC-MSCs). In contrast, UCB contains non-cellular components such as plasma and cytokines, alongside various hematopoietic and immune cell populations [19, 20]. While perinatal stem cells offer significant theoretical advantages in regenerative medicine, including low immunogenicity and high donor safety [21, 22]. Direct administration of these stem cells has shown significant potential for treating various pulmonary diseases [23–25]. However, this approach remains constrained by critical risks, such as tumorigenicity, thrombotic complications, and unintended immune responses [26, 27]. Consequently, EVs derived from these perinatal sources have emerged as a safer cell-free strategy. In this review, we categorize these vesicles based on their biological origin, distinguishing between UC-MSC-EVs and those isolated from UCB components such as plasma and immune cells, to ensure a clear understanding of their distinct therapeutic profiles.

### Umbilical cord tissue-derived EVs

This section focuses on UC-MSCs isolated specifically from umbilical cord tissue, such as Wharton’s jelly. In preclinical models, UC-MSCs have shown the potential to support the repair of damaged lung tissue, and their EVs are considered important mediators of these restorative effects [28]. These nanovesicles carry complex biological cargo, which includes approximately 304 proteins and 150 microRNAs [29]. They promote tissue repair through a variety of mechanisms, including recruiting and protecting endogenous stem cells, exerting anti-apoptotic effects, modulating immune responses, and inducing angiogenesis.

UC-MSC-EVs demonstrated therapeutic potential across multiple pulmonary injury models, encompassing scenarios including hyperoxia, lipopolysaccharide (LPS) stimulation, and even mustard gas exposure [3, 29, 30]. Their mechanism of action is multifaceted, involving a series of protective pathways: alleviating severe steroid-resistant asthma (SSRA) by promoting M2 macrophage polarization, downregulating tumour necrosis factor receptor-associated factor 1 (TRAF1) expression, and modulating NF-κB and PI3K/AKT signaling pathways [3]; countering oxidative stress by targeting caveolin-1 and redirecting miR-199a-5p to enhance NRF2 activation [30]; and mitigating LPS-induced injury by regulating m6A modification of the ITGB4 gene via EV - miR-335-5p [31].

Overall, current evidence suggests that UC-MSC-EVs are promising candidates for multi-targeted therapy. The next step is to untangle how much of their wide-ranging activity stems from the inherent heterogeneity within EV populations. To move beyond broad, phenomenological observations, we must determine which specific biological effects are produced by which distinct EVs subtypes. This can be achieved with powerful techniques like single vesicle analysis and affinity-based purification. Such approaches will be crucial for transforming UC-MSC-EVs from a promising yet variable experimental tool into a standardized and well-defined therapeutic product.

### Umbilical cord blood-derived EVs

In contrast, umbilical cord blood-derived EVs are harvested directly from the liquid plasma or specific circulating cell populations within the cord blood itself.

#### UCB-plasma-derived EVs

UCBP-EVs present significant advantages as unique therapeutic candidates, primarily due to their acellular nature and suitability for large-scale production. These EVs demonstrate pronounced protective effects in models of lung injury. For instance, UCBP-EVs exert protective effects in pulmonary injury. Following incubation with UCBP-EVs, levels of phosphorylated Akt and Erk1/2 increase in human microvascular endothelial cells. They improve fibroblast function by targeting PTEN and SPRY1 via miR-21-3p, thereby accelerating wound repair and regeneration [17]. Notably, EVs derived from healthy full-term pregnancies protect mice from hyperoxia-induced lung injury [19], directly demonstrating their potential in treating conditions such as BPD.

Although these findings are undoubtedly encouraging, the mechanistic landscape of UCBP-EVs remains only partially understood. The precise role that specific molecules (e.g., RNAs, proteins, lipids) play in pulmonary repair remains poorly defined. Future research should focus on meticulously mapping the functional cargo profiles of these vesicles and developing reliable potency assays to predict their in vivo therapeutic performance. Establishing such translational medicine benchmarks is crucial for advancing UCBP-EVs from preclinical promise to implementable clinical therapies.

#### Hematopoietic and immune cell-derived EVs

Research on UCB-HSC-EVs in lung injury is limited. This gap highlights a promising area for future study. This scarcity of data highlights not just a gap in current understanding, but also a promising opportunity for deeper investigation. UCB-HSCs possess self-renewal and multi-lineage differentiation capabilities and exhibit immunomodulatory functions, suppressing immune activation [32]. This is evidenced by their capacity to inhibit monocyte-derived dendritic cell-induced CD4⁺ T cell activation and proliferation [33], suggesting that UCB-HSC-EVs could potentially mediate these effects as paracrine messengers. However, the transition from parental cell function to definitive EVs activity is a critical, unverified step. The current evidence is largely indirect, such as findings that bone marrow MSC-EVs can influence UCB-HSCs, hinting at a broader paracrine role in microenvironmental crosstalk [33]. As a result, the essential question is not whether UCB-HSCs are immunomodulatory, but wheather UCB-HSC-EVs represent a novel, distinct therapeutic vector capable of targeted distribution to wounded lungs. This hypothesis remains compelling yet awaits rigorous experimental confirmation.

UCB contains various immune cells, such as dendritic cells, regulatory T cells (Tregs), and macrophages. These cells provide a rich source of EVs for regulating lung immunity, although more experimental research is still needed in this area [34]. These cell-associated immunoregulatory pathways reveal potent mechanisms potentially mediated via their EVs. For instance, Treg-derived EVs promote immunosuppression by inhibiting T-cell and NK-cell activity through CD73-mediated conversion of ATP to adenosine [35]. This mechanism holds promise for mitigating excessive immune activation in conditions such as acute respiratory distress syndrome. Similarly, the process of macrophage polarization towards the M2 phenotype can be regulated by EV signals such as CCL1/CCR8 signaling [36], offering another viable pathway for alleviating pulmonary inflammation.

However, the process of translating parental cell functions into lung-targeting EVs-mediated therapies remains largely speculative at present. Currently, there is limited direct evidence showing that EVs from specific UCB immune cells can reduce lung injury. This highlights the need for more studies to isolate these vesicles and confirm if they reach lung tissue and regulate the local immune environment. More experimental data are necessary to turn the potential of these immune cell-derived EVs from a hypothesis into a better understood biological mechanism.

## Therapeutic potential of UC-EVs in lung-injurious diseases

The therapeutic mechanisms of UC-EVs follow a clear functional hierarchy. They can be divided into two main categories: immunomodulation and tissue remodeling. Immunomodulation involves the regulation of inflammatory cells and cytokines, which is supported by many consistent preclinical studies. Tissue remodeling focuses on the repair of alveolar and vascular structures. While the evidence from animal models is strong for these pathways, more research is needed to confirm how stable and effective these mechanisms are in human patients. Research shows that UC-EVs have significant potential for treating different types of lung injury. These vesicles work by regulating inflammation and supporting tissue repair through the delivery of various bioactive molecules. Table 1 provides a comprehensive summary of the therapeutic effects, delivery routes, and molecular mechanisms found in recent studies. This section evaluates the current evidence for using UC-EVs across several major respiratory conditions. While some mechanisms discussed here are supported by evidence from non-pulmonary models [17, 19, 36, 37], these studies provide essential insights into the core immunomodulatory and regenerative properties of UC-EVs. These foundational processes are highly conserved across different tissues and are directly relevant to the lung environment.

**Table 1:** Summary of therapeutic effects of UC-EVs in lung-injurious diseases

References	Disease	EVs source	Therapeutic strategy	Mechanisms
[78]	BPD	hUC-MSC	IT: received 50 µl of EVs per mouse, once; for a total of 0.64 × 10^10^EVs	EVs reduced thickness of small pulmonary vessels.
[79]	BPD	hUC-MSC	In vivo: 100 µl of EVs per mouse; once; intraperitoneal injection	TSG-6 induces macrophages from proinflammatory M1 to anti-inflammatory M2 type.
[43]	BPD	UCB-Mac	In vivo: 25 mg/kg every 3 days per mouse; once; intraperitoneal injection	Silencing miR-23a-3p improved vascular damage and alveolar arrest.
[80]	BPD	UCB-Pl	In vitro: the dose of 400 µg/mL gave the optimal results at the lowest dose	EVs improved cell viability.
[45]	PF	hUC-MSC	In vitro: 20 µg/mL EVs, EVs let-7i-5p inhibitor or hucMSC-EVs-let-7i-5p NC; respectively for 24 h	Let-7i-5p-EVs relieve PF by inhibiting the turning on of fibroblasts.
[81]	PF	hUC-MSC	In vivo: 50 mg/mL EVs; per mouse; once; intratracheal instillation	EVs alleviate PF by decreasing collagen deposition in NIH-3T3 cells.
[48]	PF	hUC-MSC	In vivo; intravenous administration	MiR-218-EVs alleviate PF inhibit EndMT through MeCP2/BMP2 pathway.
[47]	IPF	hUC-MSC	In vivo: 100 µg/250 µL EVs per mouse; once; Intravenous injections	Let-7i-5p-EVs alleviate PF by inhibiting the TGF-β1/ Smad2/3 signaling pathway.
[42]	ALI	hUC-MSC	In vivo; intratracheal instillation	MiR-377-3p-EVs inhibit the expression of inflammatory factors by down-regulating mTOR.
[54]	ARDS	hUC-MSC	In vivo: 100 µl of EVs per mouse; once; intravenous injections	EVs reduce the amount of cumulative diffuse alveolar damage and levels of IL1β, IL-6 and KC.
[55]	ARDS	hUC-MSC	In vivo: 100 µl EVs per mouse; once; intravenous injections	EVs improve vascular growth by VEGF.
[82]	ALI	hUC-MSC	In vivo: 3 × 10^8^ particles; per mouse; once; tail vein injection	MiR-199a-5p activates the CAV1/NRF2 signaling pathway to reduce oxidative damage.
[83]	ARDS	hUC-MSC	In vivo: intravenous Injections	EVs enhance macrophage killing of phagocytosed *E. coli.*
[58]	Asthma	hUC-MSC	In vivo: 40 µg Hypo-EVs per mouse; once; atomisation In vitro: 40ug Hypo-EVs containing	EVs decrease the levels of albumin concomitant and enhance expression of E-cadherin and ZO-1 proteins; inhibit the decrease in E-cadherin and ZO-1, and the rise in FD4 permeability in HBE135-E6E7 cells induced by IL-4/IL-13.
[84]	Asthma	hUC-MSC	In vitro: 10,20,40 µg/ ml EVs; per mouse; once; intravenous injection	EVs regulate airway remodelling by modulating the expression of TGF-β1/Smad pathway signaling molecules.
[61]	Asthma	hUC-MSC	In vivo: 40 µg Hypo-EVs per mouse; once; atomisation	MiR-146a-5p-EVs reduce airway inflammatory cell infiltration, total cell and eosinophil counts, protein levels of ovalbumin OVA-specific IgE, IL-4, IL-5, and IL-13.
[63]	COPD	hUC-MSC	In vivo: intratracheal delivery	Protein kinase C and NF-κB subunits p65 and p50 expression alleviate pulmonary fibrosis.
[41]	COPD	hUC-MSC	In vivo: 200 µg EVs per mouse; once; tail vein injection	AKT and MEK/ERK cascade activation mediated by VEGF-VEGFR2 to prevent lung cell death.
[66]	COPD	hUC-MSC	In vivo: 15ug EVs per mouse; once; intraperitoneal injection	EVs alleviate CS-induced lung mitochondrial dysfunction.
[65]	COPD	hP-MSC	In vivo: 0.1 mL EVs per mouse; once; intraperitoneal injection	EVs rely on sTNFRI and II, IL-1Ra, and sRAGE promoting the expansion of immunosuppressive cells in the lung.

hUC-MSC, human umbilical cord-derived mesenchymal stem cell; hP-MSC, human placenta-derived mesenchymal stem cell; UCB-Pl, umbilical cord blood-derived plasma; UCB-Mac, umbilical cord blood-derived macrophage; EVs, extracellular vesicles; BPD, bronchopulmonary dysplasia; PF, pulmonary fibrosis; ARDS, acute respiratory distress syndrome; ALI, acute lung injury; COPD, chronic obstructive pulmonary disease; IT, intratracheal; LPS, lipopolysaccharide

### Bronchopulmonary dysplasia

BPD is a major respiratory complication in preterm infants [38], and is characterized pathologically by impaired alveolarization and compromised vascular development [39]. Current approaches, such as supplemental oxygen and mechanical ventilation, can reduce injury but fail to reverse developmental arrest [40], thereby creating a compelling therapeutic opportunity for UCB-EVs.

The therapeutic potential of UC-EVs in BPD is demonstrated by their mechanisms of action targeting core pathological processes. In hyperoxia models, UC-EVs activate the AKT and MEK/ERK pathways via vascular endothelial growth factor receptor 2 (VEGFR2) [41], thereby maintaining endothelial integrity and directly promoting angiogenesis. Concurrently, they enhance protective autophagy through miR-377-3p-mediated mTOR inhibition, thereby improving lung histology and reducing inflammatory mediators in bronchoalveolar lavage fluid [42]. Furthermore, the roles of specific miRNAs in EVs are being studied. For example, research showed that silencing miR-23a-3p in EVs from UCB macrophages can reduce vascular injury and help maintain alveolar structure [43].

These preclinical findings suggest that UC-MSC-EVs represent a potential therapeutic approach for managing lung developmental arrest. The significant difficulty now lies in identifying the optimal EVs source and determining the therapeutic window that best aligns with the pathophysiological characteristics of lung dynamics in preterm infants. Optimizing these parameters is important for turning current research results into potential therapeutic strategies for neonatal respiratory diseases.

### Pulmonary fibrosis

PF is a chronic progressive interstitial lung disease characterized by excessive deposition of extracellular matrix and scar tissue, leading to irreversible decline in pulmonary function [44].

UC-MSC-EVs demonstrate significant anti-fibrotic potential by targeting core pathogenic pathways. Their primary mechanism involves direct inhibition of the transforming growth factor-β (TGF-β) signaling axis. Let-7i-5p within UC-MSC EVs suppresses fibroblast activation via the TGF-β1/Smad3 pathway [45], while broader EVs preparations have been demonstrated to reverse epithelial-mesenchymal transition (EMT) by inhibiting both TGF-β1/Smad2/3 and Wnt/β-catenin dual signaling pathways [46, 47]. Furthermore, EV-miR-218 inhibits endothelial-to-mesenchymal transition (EndMT) via the MeCP2/BMP2 axis [48], thereby regulating another important factor in the fibrotic microenvironment. The therapeutic potential of EVs can be further improved by genetically modifying their source cells. For example, EVs derived from UC-MSCs that overexpress hepatocyte growth factor have shown much stronger anti-fibrotic effects in pulmonary fibrosis models [49].

It is noteworthy that the role of EVs in pulmonary fibrosis is complex and context-dependent. Contrary to the therapeutic effects of UC-MSC-EVs, certain EVs may worsen fibrosis; for instance, studies indicate that upregulation of EV-miR-34a accelerates pulmonary epithelial cell senescence and apoptosis, thereby driving fibrosis progression in mouse models [50].

A growing body of evidence conclusively demonstrates that UC-MSC-EVs, as a multi-mechanistic therapeutic approach, holds significant promise for pulmonary fibrosis. However, to fully realize its clinical potential, the duality of EV activity within this disease must be addressed. Future research should precisely identify the molecular payloads underlying therapeutic versus pathogenic effects and validate these mechanistic findings in humanized or physiologically relevant progressive fibrosis models. Such work is crucial for optimizing EV interventions into safe, predictable therapies that genuinely alter disease progression.

### Acute respiratory distress syndrome/acute lung injury

ARDS and its milder counterpart, ALI, are vital conditions characterized by uncontrolled pulmonary inflammation and disruption of the alveolar-capillary barrier [51], leading to profound gas exchange impairment [52].

UCB-EVs demonstrate multidimensional therapeutic efficacy in ARDS/ALI by targeting key inflammatory and injury pathways. They reduce excessive inflammatory responses by delivering miR-451 to modulate the TLR4/NF-κB pathway, significantly reducing TNF-α, IL-1β, and IL-6 levels in burn-induced ALI models [53]. Similarly, by downregulating mTOR expression via miR-377-3p, they not only inhibit inflammatory mediator production but also activate protective autophagy [42]. Their key mechanism lies in regulating neutrophil-driven injury: UCB-MSC-EVs simultaneously inhibit neutrophil migration and neutrophil extracellular trap formation while transferring mitochondrial manganese superoxide dismutase to alleviate oxidative stress [37]. Consistent with these effects, intravenous administration of UCB-EVs reduces markers of diffuse alveolar damage while promoting an anti-inflammatory macrophage phenotype characterized by elevated Arg1, Ym-1, and CD206 expression [54, 55].

These findings suggest that UCB-EVs hold great promise as a multi-targeted therapy addressing the complex pathophysiology of ARDS/ALI. The primary challenge now lies in translating this broad efficacy into a standardized biological preparation, requiring future studies to identify the most active EV subpopulations and validate their safety and efficacy in physiologically relevant, larger animal models.

### Asthma

Asthma is a heterogeneous chronic airway disease characterized by inflammation, remodeling, and variable airflow obstruction [56]. Standard treatments, such as corticosteroids, are often not effective for SSRA and can lead to side effects after long-term use [30].

UCB-EVs demonstrate therapeutic efficacy in SSRA models by reducing lung inflammation and airway hyperresponsiveness via modulation of NF-κB and PI3K/AKT signaling. Mechanistically, UCB-EVs target TRAF1 to alter macrophage polarization and attenuate inflammatory cascades [30]. Additionally, airway epithelial barrier damage is a key feature of asthma [57]. Recent evidence shows that UCB-EVs can help restore barrier integrity [58]. This repair mechanism suggests that nebulized UCB-EVs might also provide benefits for other conditions with similar barrier damage, such as COPD and chronic rhinitis. Type 2 inflammation in asthma features IL-4 and IL-13 secretion from Th2 cells [59], while TGF-β1 acts as a potent chemokine promoting inflammatory cell infiltration, highlighting its complex role in inflammation regulation [60]. In preclinical studies, nebulized hypoxic UCB-EVs significantly reduce inflammatory parameters with a favorable safety profile, decrease total cell/eosinophil counts, ovalbumin-specific IgE, IL-4, IL-5, IL-13 levels, and airway inflammatory infiltration [61]. Although nebulization is a viable delivery method, more research is needed to understand the specific target cells and molecular mechanisms of EV inhalation therapy.

### Chronic obstructive pulmonary disease

COPD is a prevalent respiratory disorder characterized by persistent airflow limitation and chronic inflammation, typically induced by inhalation of harmful particulate matter such as cigarette smoke [62].

UCB-EVs demonstrate potential in alleviating multiple pathological features of COPD. Research shows they can target major signaling pathways in COPD, such as NF-κB, IL-4, and TGF-β receptor signaling [63]. A crucial protective mechanism involves preserving lung architecture by activating VEGF-VEGFR2-mediated AKT and MEK/ERK pathways, thereby preventing emphysematous cell death [64]. In preclinical models of cigarette smoke exposure, MSC-derived EVs improve lung function by reducing inflammatory cytokine levels and decreasing the number of infiltrating immune cells in the lungs, while simultaneously increasing the anti-inflammatory factor IL-10 and the number of Tregs [65]. Furthermore, they protect against cigarette smoke-induced mitochondrial dysfunction [66], thereby ameliorating a key pathological feature of COPD.

However, the role of EV in COPD is not exclusively beneficial. Notably, EV *hsa_circ_0005045* upregulation has been implicated in exacerbating COPD pathology through PRDX2/ELANE/TNF-α pathway activation [67]. This variability further indicates that not all EVs possess inherent therapeutic efficacy; indeed, EVs derived from certain cell sources may exert harmful or even pathogenic effects. Such variability highlights the imperative to rigorously validate sources and characterize cargo composition prior to establishing clinical efficacy.

However, it is necessary to evaluate the quality of these preclinical studies. Most research in this field relies on small animal models, which may not fully represent the complexity of human lung diseases. There are also clear differences in how different laboratories induce lung injury and decide on the dose and timing of EV treatment. Many studies have small sample sizes and do not compare EVs from different sources directly. These issues make it difficult to repeat the results or compare findings across different papers. Therefore, more standardized research is needed to confirm the true therapeutic value of these vesicles before moving to clinical trials.

## Future challenges and directions

### Engineering strategies for improving treatment

**Fig. 3:**
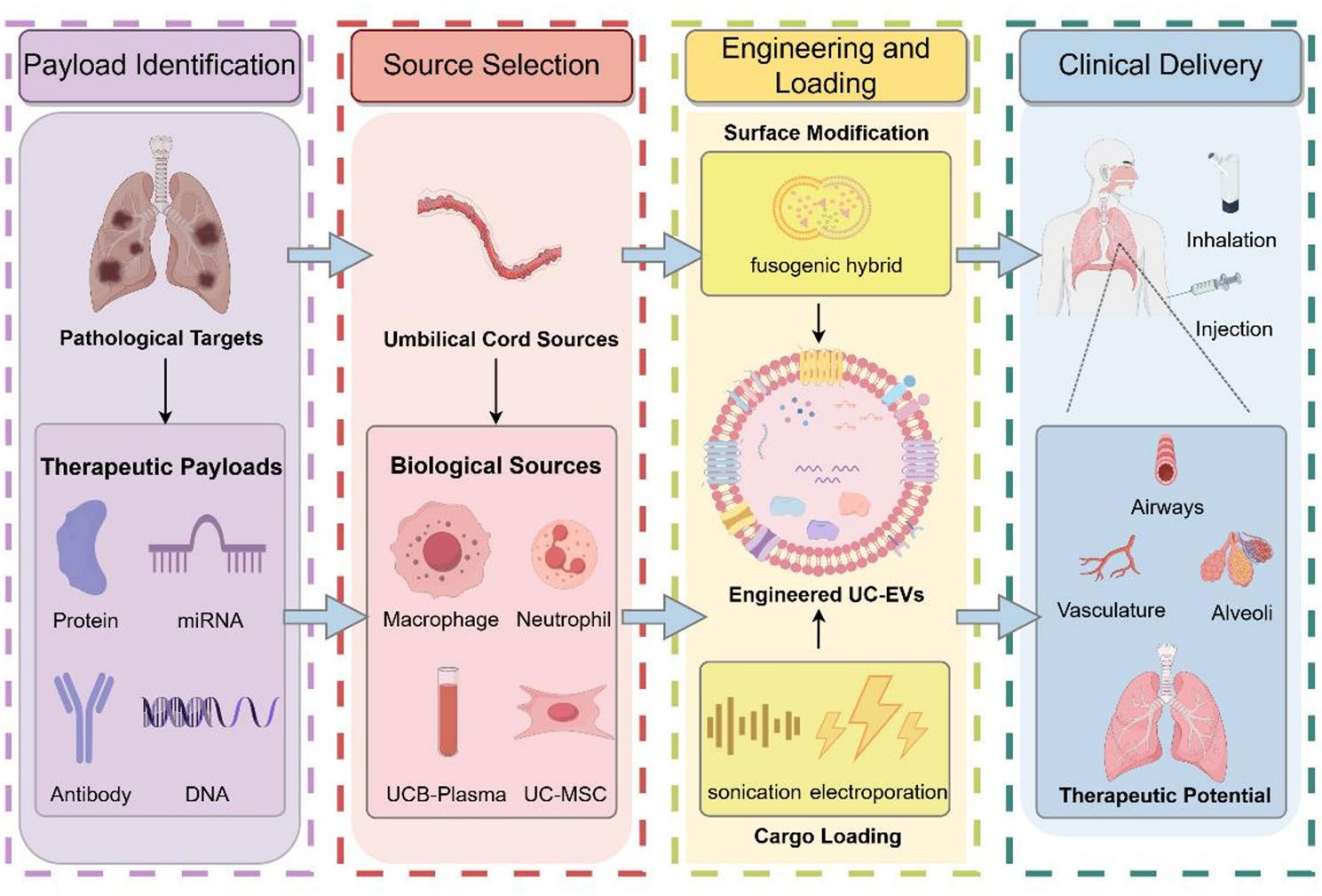
Translational framework for the design and delivery of engineered UC-EVs. This figure illustrates the systematic pipeline for creating engineered UC-EVs for lung therapy. The process begins with Payload Identification, where specific pathological targets in the lung drive the selection of therapeutic molecules such as proteins, miRNAs, antibodies, and DNA. Next, Source Selection involves choosing the most appropriate biological materials from the umbilical cord, including tissue-derived UC-MSCs and blood-derived components like plasma and immune cells

The logical hierarchy for developing engineered EVs, from payload identification to clinical implementation, is summarized in Fig. 3. The potential of UC-EVs can be improved through engineering methods to help lung repair. By choosing the right cargo, modifying the source cells, and selecting the delivery route, these EVs can be made more specific for lung injury. Research has focused on loading molecules like miR-199a-5p and mRNA or functional proteins [30] to regulate specific repair mechanisms. External modification is also an important factor that influences the natural activity and targeting of EVs. While the umbilical cord is an excellent source, cells can also be modified to express lung-targeting peptides on the EV surface [68] to help them reach injury sites. Common loading methods include electroporation and ultrasonication [69], but these must not damage the EV structure or its natural function. For delivery, nebulization allows EVs to reach the airways and alveoli directly [70], while intravenous injection is better for targeting the lung vasculature [71].

### Scaling production and ensuring consistency

Scaling up production is a major challenge for clinical use. Laboratory methods like ultracentrifugation are not practical for large-scale manufacturing. New methods like cellular nanoporation can produce many EVs with mRNA [72], but these must be optimized for umbilical cord sources to maintain efficiency and quality. A significant bottleneck is the variability between different batches. Differences in donors, cell passage numbers, and culture conditions can change the cargo and activity of EVs. Therefore, standardized protocols for donor screening and cell culture are necessary to ensure that every batch is consistent [14].

Safety is also a major concern. As discussed in the sections on specific lung diseases, not all UC-EVs are inherently safe. Evidence shows that certain EV subpopulations from diseased or stressed environments can be pathogenic and may even worsen lung injury [73]. This variability highlights the need for strict quality control and thorough safety testing of every EV source before clinical translation.

### Clinical translation and future perspectives

Although Zofin is derived from amniotic fluid rather than the umbilical cord, its successful application in COVID-19 cases provides important preliminary evidence for the safety of perinatal tissue-derived EVs [74]. Currently, several registered clinical trials are specifically investigating the safety and potential of UC-MSC-EVs for treating pulmonary conditions such as ARDS and BPD. The details of these registered clinical trials are summarized in Table 2. To move forward, we must establish Good Manufacturing Practice standards [75]. Crucially, we need to develop functional potency assays. Measuring the size and concentration of EVs is not enough to guarantee their biological effect. We must use specific biological tests to prove that each batch of EVs can actually reduce inflammation or promote repair before it is given to patients [76]. New tools like single-vesicle analysis and advanced imaging [77] will help us understand how EVs behave in the body. By combining better manufacturing with these insights, UC-EVs can move from research toward becoming a real treatment for lung diseases.

**Table 2:** Registered clinical trials involving UC-EVs in lung-injurious diseases

Identifier	Disease	EVs source	Status	Year	Intervention
ChiCTR2400091226	ARDS	hUC-MSC	Recruiting	2024	Inhalation
ChiCTR2300069181	COVID-19	hUC-MSC	Completed	2023	Inhalation
ChiCTR2300075466	PF	hUC-MSC	Recruiting	2023	Inhalation
ChiCTR2000030261	COVID-19	hUC-MSC	Completed	2020	Inhalation
ChiCTR2000030484	COVID-19	hUC-MSC	Completed	2020	IV
NCT05787288	COVID-19	hUC-MSC	Recruiting	2023	Inhalation
NCT05387278	ARDS	hUC-MSC	Recruiting	2022	IV
NCT06279741	BPD	hUC-MSC	Recruiting	2023	ETP instillation

ARDS, acute respiratory distress syndrome; BPD, bronchopulmonary dysplasia; COPD, chronic obstructive pulmonary disease; COVID-19, coronavirus disease 2019; EVs, extracellular vesicles; hUC-MSCs, human umbilical cord-derived mesenchymal stem cells; PF, pulmonary fibrosis; IV, intravenous; ETP, endotracheopulmonary

The clinical development of UC-EVs for lung diseases is still in its early stages. Most human studies are small-scale Phase I or Phase II trials. The primary goal of these studies is to evaluate safety and feasibility rather than to prove clinical efficacy. Currently, there are no Phase III randomized controlled trials to confirm that UC-EVs can effectively treat these conditions. Therefore, while early results are positive, more large-scale studies are needed before these therapies can be used in routine clinical practice.

## Conclusion and future perspectives

Lung-injurious diseases remain difficult to treat because the way they develop is very complex. UC-EVs are a promising strategy because they can help with immune regulation, reduce fibrosis, and support tissue repair. However, it is important to remember that most current evidence comes from animal studies. While early human studies show that these EVs are safe, their clinical effectiveness has not yet been proven in Phase III randomized controlled trials.

To reach the full potential of UC-EVs, future research must focus on finding the most active vesicles and improving how they reach the lungs. We also need to solve production challenges, such as making sure every batch is the same quality and developing tests to check their strength. More clinical studies are needed to confirm safety and long-term results. While UC-EVs are a strong biological concept, more rigorous work is needed to turn them into real treatments for patients.

The Engineering and Loading stage represents the core manufacturing process. It is categorized into surface modification and internal cargo loading (e.g., sonication and electroporation) to produce the final engineered UC-EVs. Finally, the Clinical Delivery stage details the administration of these vesicles through intravenous injection or nebulized inhalation to target specific areas of the lung, including the airways, alveoli, and vasculature, to achieve their therapeutic potential. This diagram was professionally designed and generated by the authors via Figdraw (www.figdraw.com).

## Data Availability

No datasets were generated or analysed during the current study.
